# 651. Implementing a Multiplex-PCR Test for the Diagnosis of Acute Gastroenteritis in Hospitalized Children, Are All Enteric Viruses the Same?

**DOI:** 10.1093/ofid/ofad500.714

**Published:** 2023-11-27

**Authors:** Dana Danino, Guy Hazan, Rufaida Mahajna, Firas Khalde, Lama Farraj, Yonat Shemer-Avni, David Greenberg, Eli Hershkovitz, Noga Givon-Lavi, Yaniv Faingelernt

**Affiliations:** Soroka University Medical Center, Pediatric Infectious Disease Unit, Beer Sheva, HaDarom, Israel; Soroka University Medical Center, Pediatric Department D, Beer Sheva, HaDarom, Israel; Soroka University Medical Center, Pediatric Dpartment D, Beer Sheva, HaDarom, Israel; Soroka University Medical Center, Pediatric Department D, Beer Sheva, HaDarom, Israel; Soroka University Medical Center, Pediatric Department D, Beer Sheva, HaDarom, Israel; Soroka University Medical Center, Clinical Virology Laboratory, Beer Sheva, HaDarom, Israel; Soroka University Medical Center, Pediatric Infectious Disease Unit, Beer Sheva, HaDarom, Israel; Soroka University Medical Center, Pediatric Infectious Disease Unit, Beer Sheva, HaDarom, Israel; Soroka Univ Med Ctr and Ben-Gurion Univ, Beer Sheva, HaDarom, Israel; Soroka University Medical Center, Pediatric Gastroentereology, Hepatology and Nutrition Unit, Beer Sheva, HaDarom, Israel

## Abstract

**Background:**

Multiplex-PCR for the diagnosis of acute gastroenteritis (AGE) has allowed for rapid detection of multiple pathogens simultaneously but poses challenges in distinguishing between shedding and disease-causing pathogens. We aimed to evaluate the epidemiology of viral AGE and compare clinical characteristics among the 5 most common viruses.

**Methods:**

SUMC is the only hospital in southern Israel serves a population of ∼97,500 children < 5 years. Rotavirus vaccine was added to the national immunization program in 2010 and vaccine coverage (3 doses) reached 70% in 2 years. All rectal swabs for multiplex-PCR targeting; rotavirus, norovirus, adenovirus 40,41, astrovirus and sapovirus collected from diarrheic children < 5 years, December 2017 - March 2022, were included. Detection of the same virus within 2 months was considered a single episode. The clinical analysis included all episodes with single-virus detection and negative stool bacterial PCR **(Figure 1)**.

**Figure:**
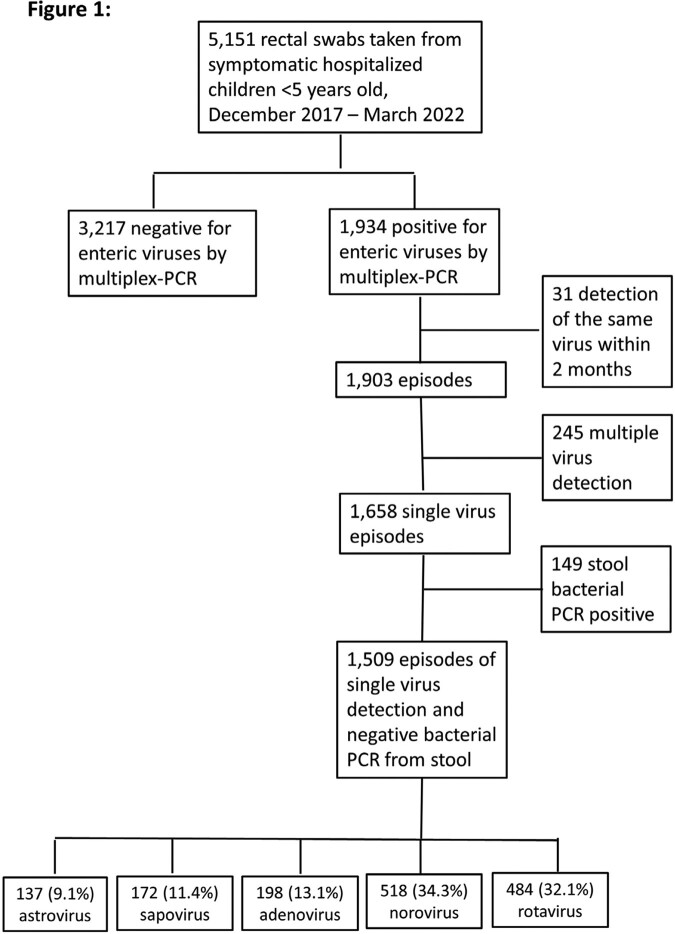

**Results:**

Of the 5,151 rectal swabs collected, 1,934 (37.5%) were positive for at least one virus, with 245 (12.6%) showing multiple virus detection. Norovirus and rotavirus were the most prevalent **(Figure 1)**. No clear seasonality was observed for AGE viruses in 2018-19. However, during the 2^nd^ year of the COVID-19 pandemic, an unusual surge in warm months was observed, first attributed to rotavirus and later to norovirus **(Figure 2)**. Of the single-virus AGE episodes with negative bacterial PCR, 34.6% and 5.9% presented with mucus and bloody stool, respectively, and 29.3% were treated with antibiotics. No significant differences in rotavirus vaccination rates were found between rotavirus and other viruses. Children infected with astrovirus and sapovirus had higher rates of hospital-acquired AGE and immunodeficiency (P< 0.05) **(Table)**, while children with rotavirus had higher dehydration severity and metabolic acidosis (P< 0.05) **(Figure 3)**.

**Figure:**
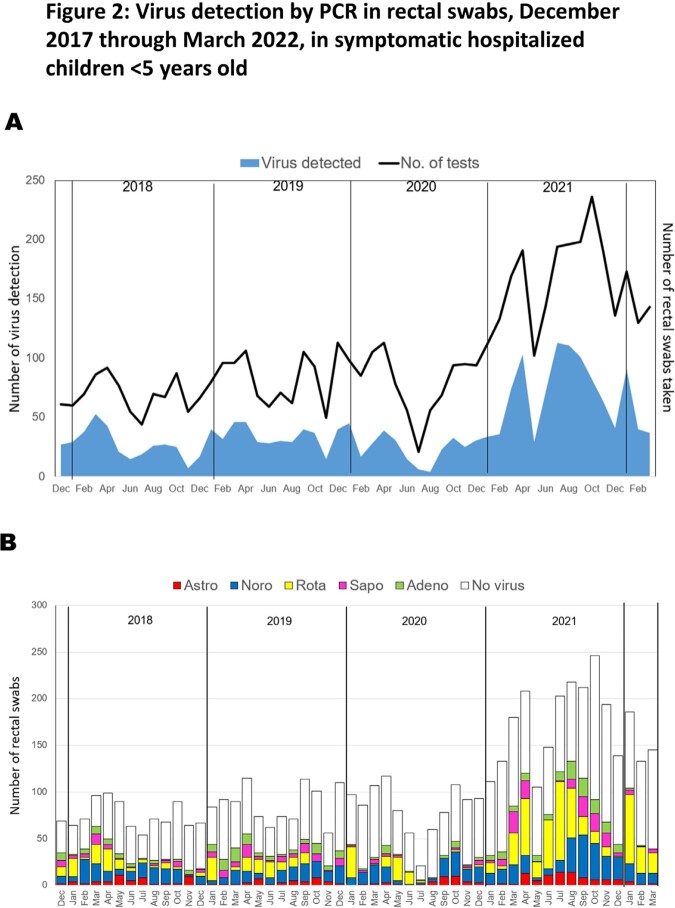

**Figure:**
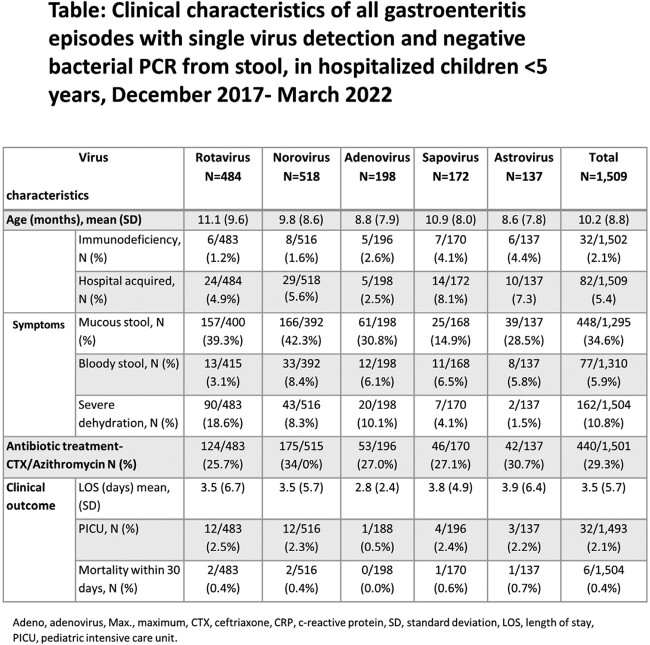

**Figure:**
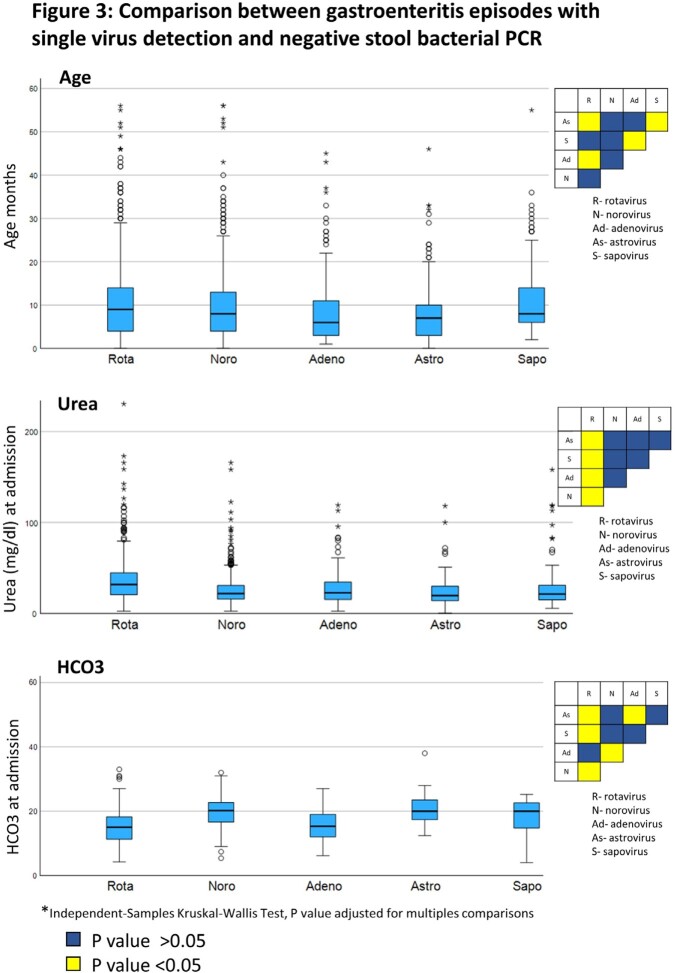

**Conclusion:**

37.5% of all rectal swabs from diarrheic hospitalized children < 5 years were positive for enteric virus, 12.6% with multiple detection. Rotavirus remained significant and the most severe despite vaccination efforts. Our findings highlight the importance of continuous surveillance in the context of multiplex PCR testing for accurate management and future prevention methods.

**Disclosures:**

**Dana Danino, Dr. MD**, Pfizer: Grant/Research Support **David Greenberg, Professor MD**, GSK: Advisor/Consultant|GSK: Honoraria|MSD: Advisor/Consultant|MSD: Grant/Research Support|MSD: Honoraria|Pfizer: Advisor/Consultant|Pfizer: Honoraria

